# Supplementary material for: A room-temperature sodium–sulfur battery with high capacity and stable cycling performance
Source: Nat Commun. 2018 Sep 24;9:3870. doi: 10.1038/s41467-018-06443-3 (PMC6155237; doi:10.1038/s41467-018-06443-3)
Supplement: Supplementary file 1 — Supplementary Information [file 41467_2018_6443_MOESM1_ESM.pdf]

Supplementary Information

A room-temperature sodium-sulfur battery with high capacity and stable cycling performance

Xu et al.

Supplementary Figures

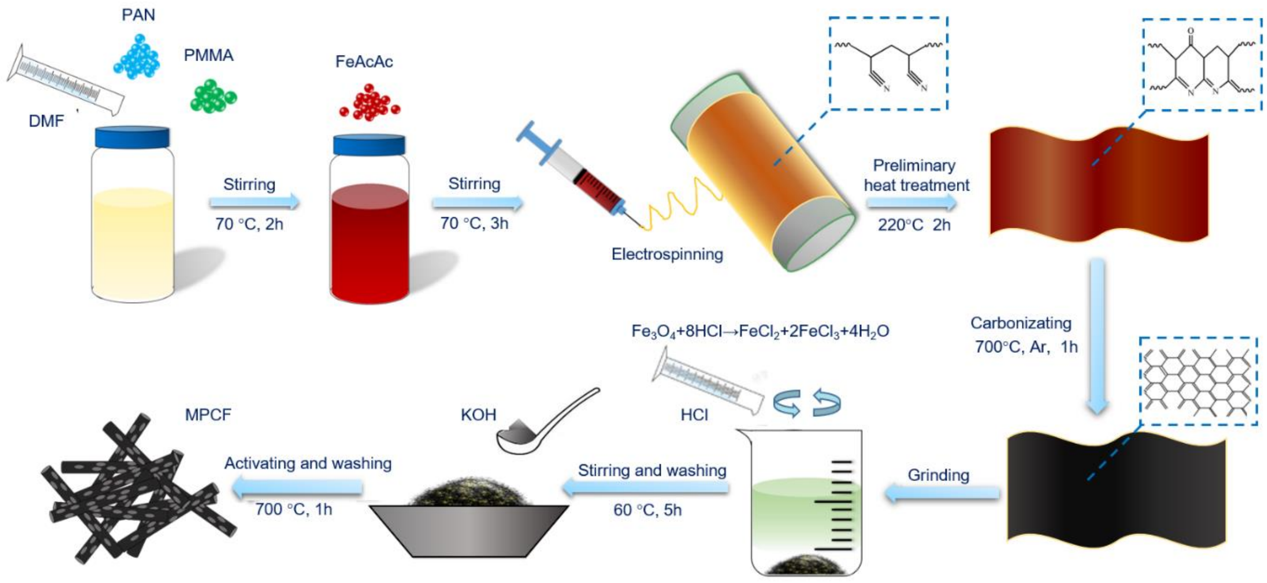

Supplementary Figure 1 Schematic illustration of the preparation process for multiporous carbon fibers (MPCFs).

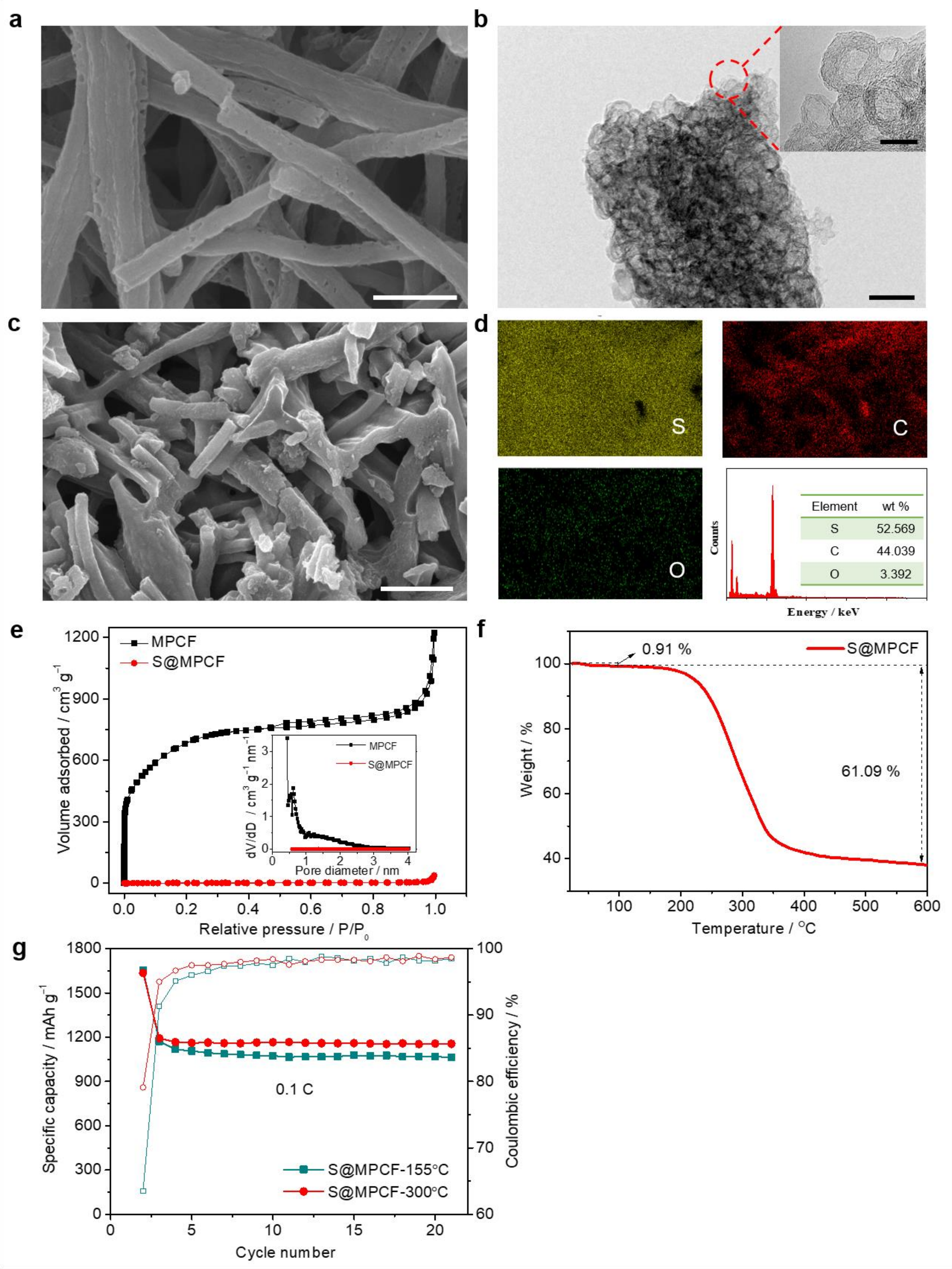

Supplementary Figure 2 Characterization of MPCF and S@MPCF. **a** SEM (scale label is 500 nm) and **b** TEM (scale label is 50 nm, and 10 nm in the insert) images of MPCF; **c** SEM image (scale label is 1 μm) and **d** EDS mappings of sulfur, carbon, oxygen and EDS spectrum of S@MPCF (the percentages of elements are shown in inset); **e** N_2 adsorption-desorption isotherms

and corresponding Barrett-Joyner-Halenda (BJH) pore size distributions (shown in inset) of MPCF and S@MPCF; **f** TGA curves of the S@MPCF under N_2 flow from 20 to 600 ^{o}C at a heating rate of 10 ^{o}C min^{-1}; **g** Cycling performances of Na/2 M NaTFSI in PC: FEC (1: 1 by volume) with 10 mM InI_3/S@MPCF cells at 0.1 C, using S@MPCF powders obtained by heating the S/MPCF mixture at 155 ^{o}C with or without a further heating treatment at 300 ^{o}C.

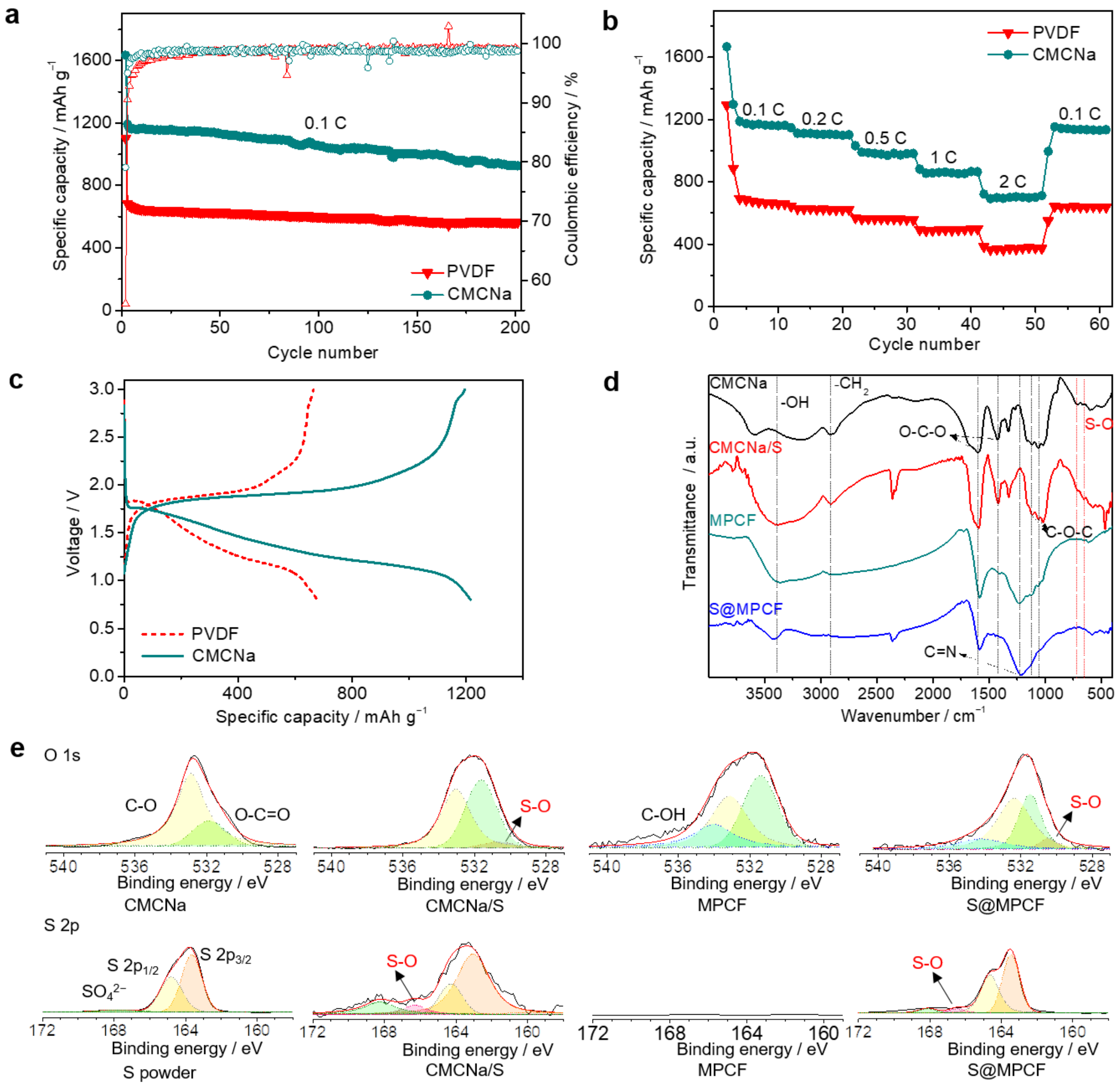

Supplementary Figure 3 The effect of different binders on the electrochemical performances. a Cycling performances, **b** rate performances and **c** 5th charge/discharge profiles at 0.1 C of Na/2 M NaTFSI in PC: FEC (1: 1 by volume) with 10 mM InI_3/S@MPCF cells using CMCNa or PVDF as binders; **d** FTIR spectra of CMCNa, CMCNa/S mixture, MPCF and S@MPCF; **e** XPS spectra of O 1s and S 2p for sulfur, CMCNa, CMCNa/S mixture, MPCF and S@MPCF.

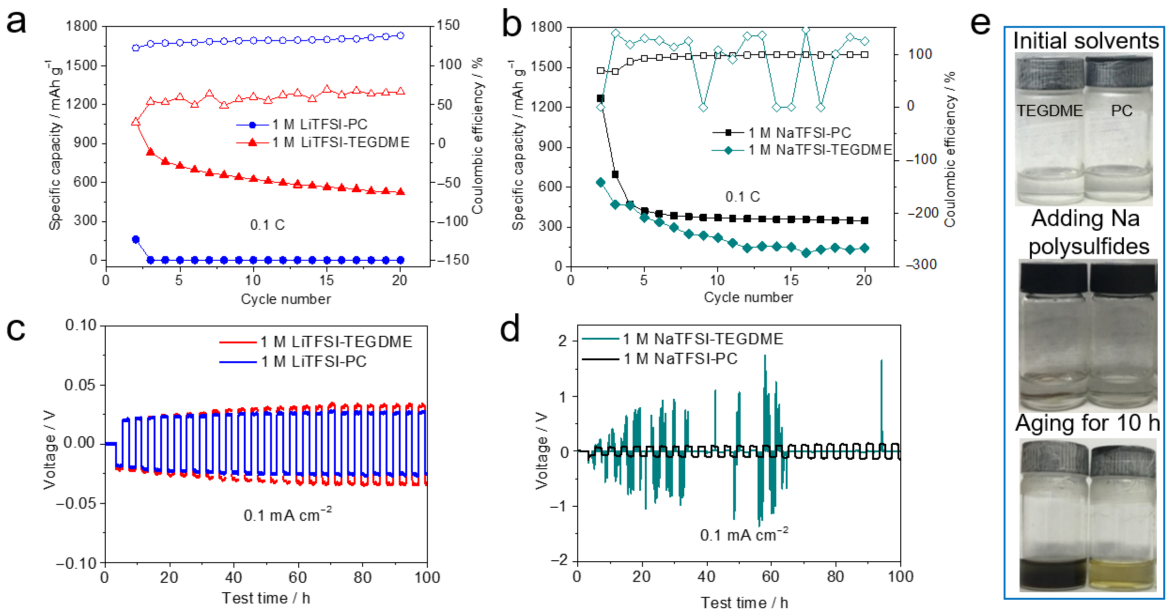

Supplementary Figure 4 Electrochemical characterization of Na-S and Li-S batteries in TEGDME-based and PC-based electrolytes. The cycling performances of **a** Li/S@MPCF batteries and **b** Na/S@MPCF batteries using 1 M LiTFSI (or NaTFSI) in TEGDME and 1 M LiTFSI (or NaTFSI) in PC electrolytes at 0.1 C. Galvanostatic cycling curves of **c** Li/Li and **d** Na/Na symmetrical cells in TEGDME-based and PC-based electrolytes at current density of 0.1 mA cm^{-2}; **e** The color changes of TEGDME and PC solvents containing the same amount of Na polysulfides recorded by digital camera along with time of sampling.

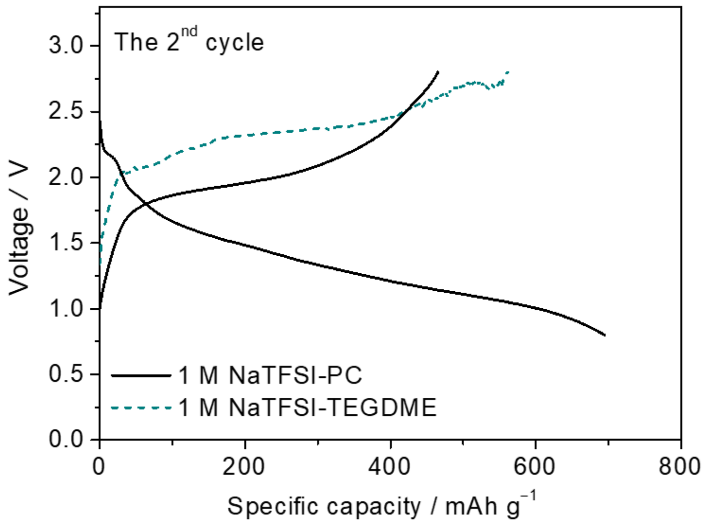

Supplementary Figure 5 The 2nd charge/discharge profiles of Na/S@MPCF cells with 1 M NaTFSI in PC or 1 M NaTFSI in TEGDME electrolytes at 0.1 C.

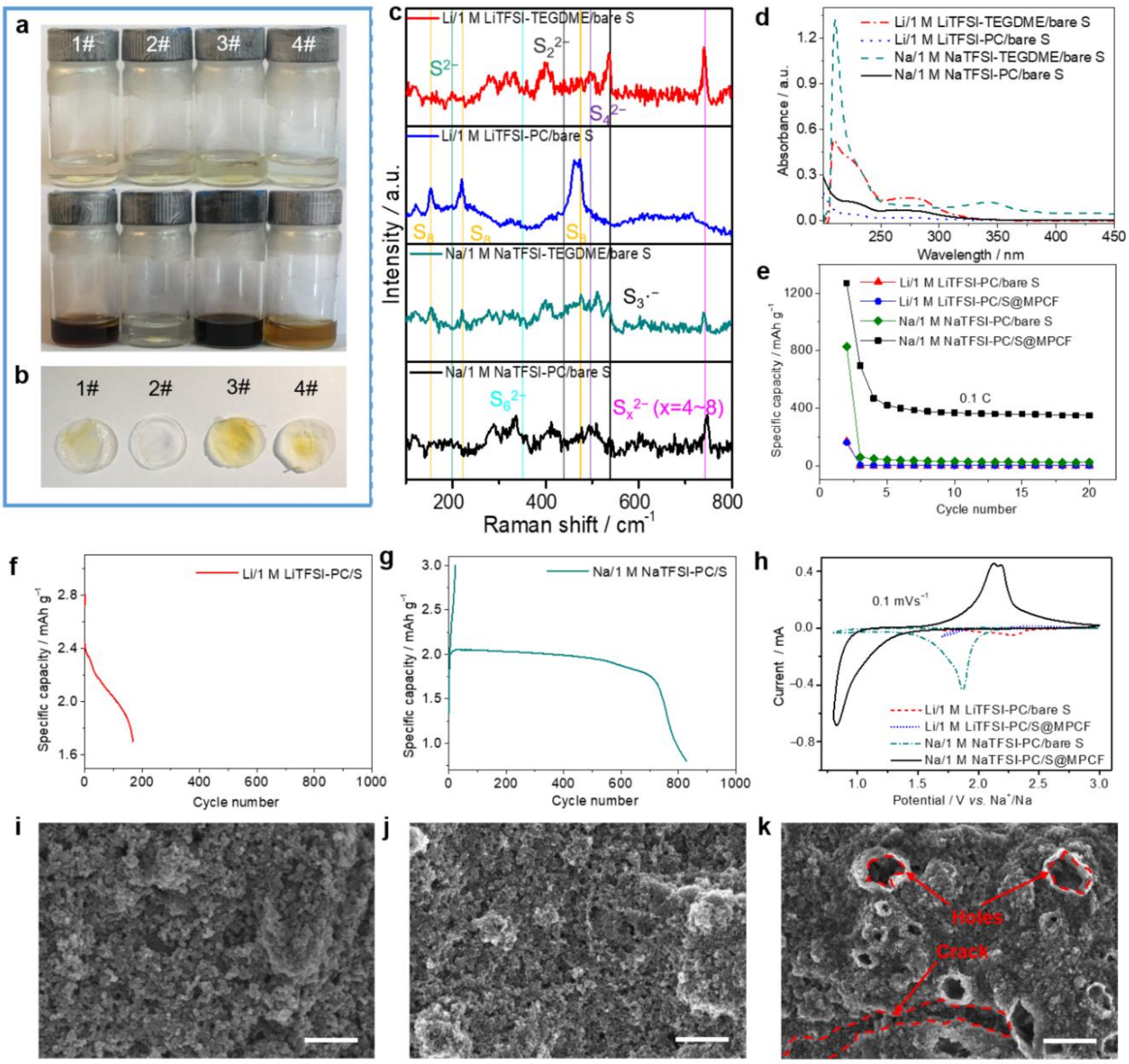

Supplementary Figure 6 The reactions between polysulfide intermediates and carbonate solvents. a Visual observation of polysulfides formation in four electrolyte samples (1#:1 M LiTFSI in TEGDME with Li metal foil; 2#: 1 M LiTFSI in PC with Li metal foil; 3#: 1 M NaTFSI in TEGDME with Na metal foil; 4#: 1 M NaTFSI in PC with Na metal foil) along with aging at 60 ^{o}C for 48 h. The same amounts of sulfur (10 mg) were added into 3 g electrolyte samples to simulate the self-discharge processes. **b** The optical images of the segregators obtained from 1#: Li/1 M LiTFSI in TEGDME/bare S; 2#: Li/1 M LiTFSI in PC/bare S; 3#: Na/1 M NaTFSI in TEGDME/bare S; 4#: Na/1 M NaTFSI in PC/bare S cells after the initial discharging at 0.1 C. **c** Raman spectra of the bare sulfur electrodes obtained from Li/1 M LiTFSI in TEGDME/bare S, Li/1 M LiTFSI in PC/bare S, Na/1 M NaTFSI in TEGDME/bare S and Na/1 M NaTFSI in PC/bare S cells after an initial discharging at 0.1 C. **d** UV-Vis spectra of different electrolyte samples with same amounts of sulfur powder and Li or Na metal foils after aging at 60 ^{o}C for 48 h, corresponding to Supplementary Fig. 6a. S_4^{2-}: ~320 nm; S^{2-} and S_2^{2-}: 220~260 nm[1].

e The cycling performances of Na-S and Li-S batteries using bare sulfur cathode or S@MPCF cathode in PC-based electrolytes at 0.1 C, and **h** the corresponding CV curves at a scan rate of 0.1 mV s^{-1}; **f** The initial charge/discharge profiles of a Li/1 M LiTFSI in PC/bare S and **g** Na/1 M NaTFSI in PC/bare S cells at 0.1 C; The FE-SEM images of **i** the initial bare sulfur electrode, the bare sulfur electrodes obtained from **j** Li/1 M LiTFSI-TEGDME/bare S and **k** in Na/1 M NaTFSI-PC/bare S cells after an initial discharging at 0.1 C, Scale bars are 2 μm in Supplementary Fig. 6i–k.

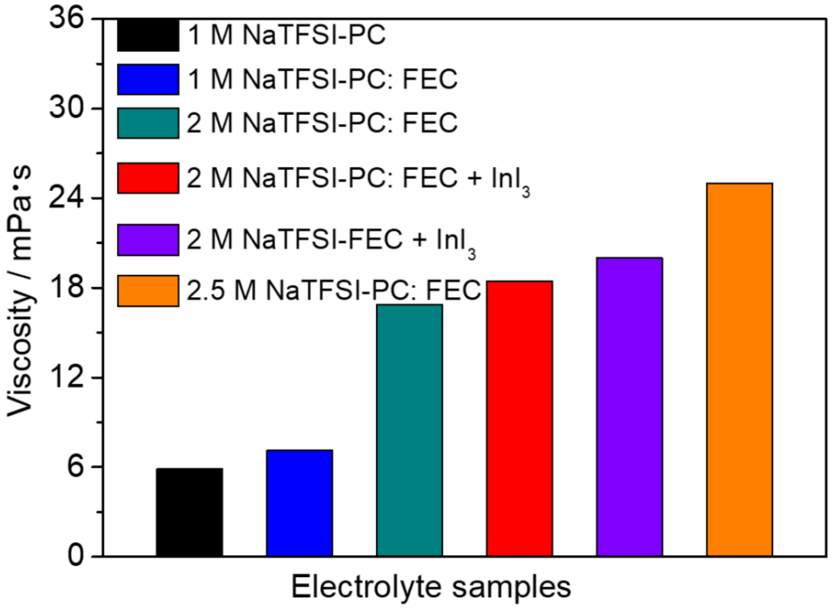

Supplementary Figure 7 The viscosities of 1 M NaTFSI in PC, 1 M NaTFSI in PC: FEC (1: 1 by volume), 2 M NaTFSI in PC: FEC (1: 1 by volume), 2 M NaTFSI in PC: FEC (1: 1 by volume) with 10 mM InI_3, 2 M NaTFSI in FEC with 10 mM InI_3 electrolytes and 2.5 M NaTFSI in PC: FEC (1: 1 by volume) electrolytes at 25 ^oC.

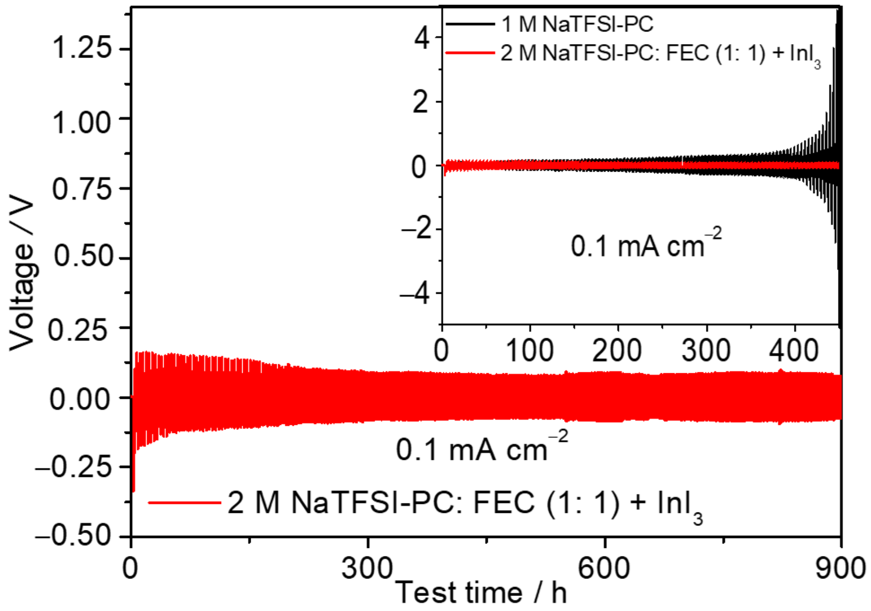

Supplementary Figure 8 Galvanostatic cycling curves of Na/Na symmetrical cells using 1 M NaTFSI in PC and 2 M NaTFSI in PC: FEC (1: 1 by volume) with 10 mM InI_3 electrolytes.

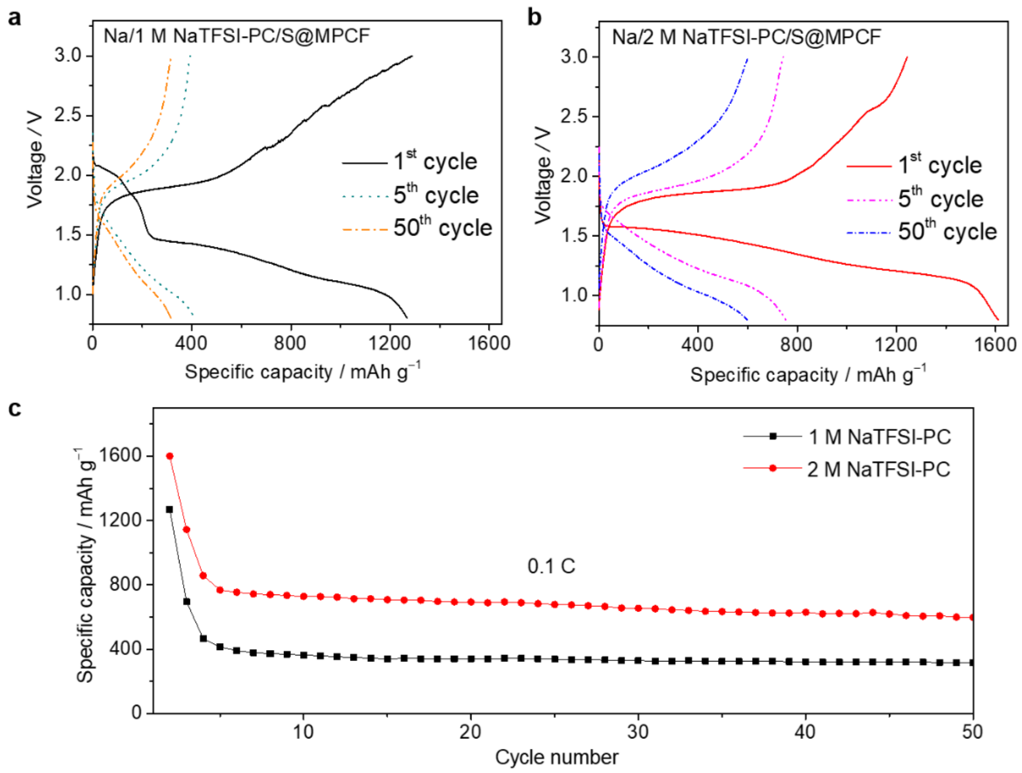

Supplementary Figure 9 Electrochemical performances of Na/S@MPCF cells applying different concentrations of salt in electrolytes. The 1st, 5th and 50th charge/discharge profiles of Na/S@MPCF cells using **a** 1 M NaTFSI in PC and **b** 2 M NaTFSI in PC electrolytes at 0.1 C; **c** The cycling performances of Na/S@MPCF cells using different electrolytes at 0.1 C. When the salt concentration of the electrolyte is increased from 1 M to 2 M, the reversible capacity of the Na/2 M NaTFSI in PC/S@MPCF cell significantly improves from 314 mAh g^{-1} to 589 mAh g^{-1} after 50 cycles at 0.1 C.

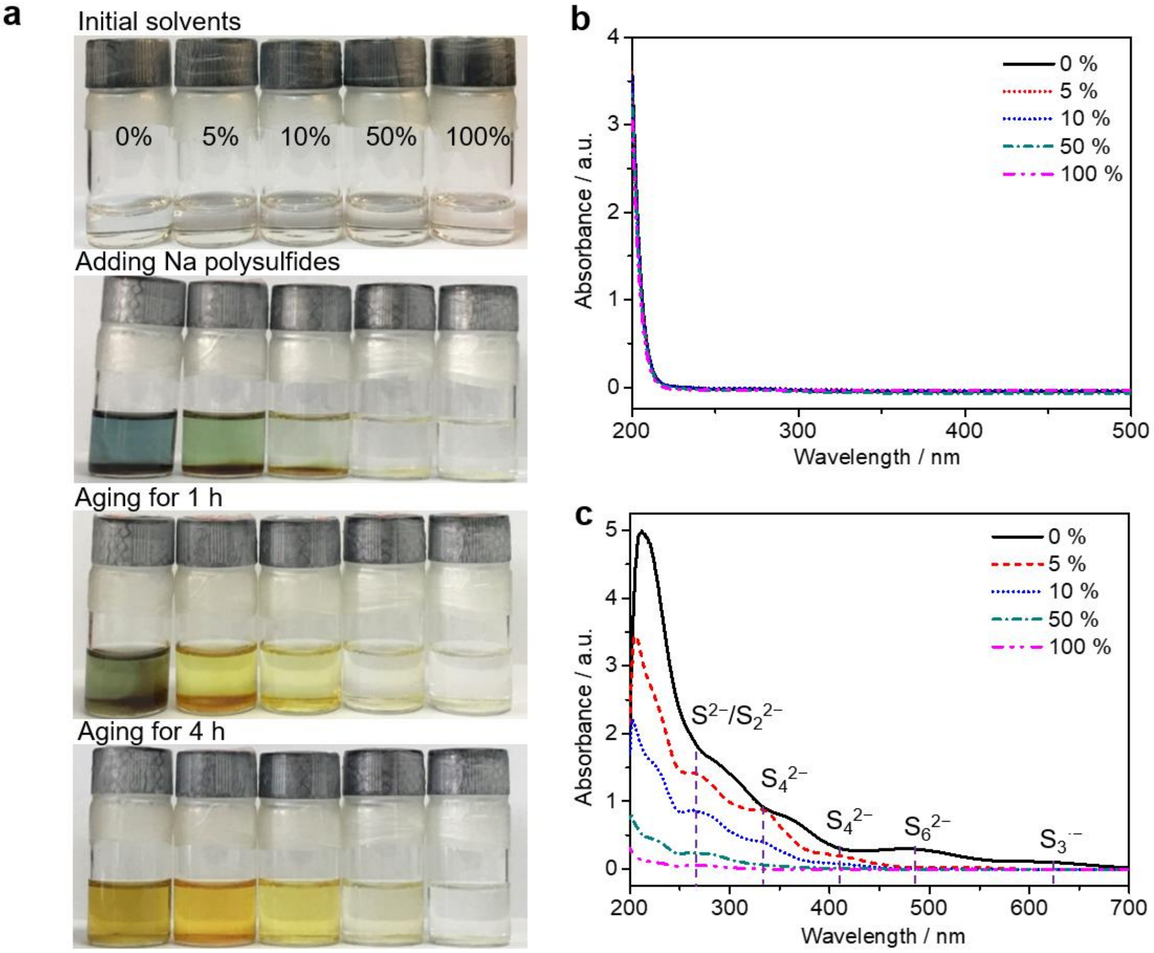

Supplementary Figure 10 The formation of Na polysulfide in solvents with different FEC proportions. a Optical images of PC: FEC mixtures with various FEC proportions and 5 mg added Na_2S_8 along with aging time at 60 ^oC. The corresponding UV-Vis spectra of **b** initial PC: FEC solvents with various FEC proportion and **c** PC: FEC solvents with Na_2S_8 after aging for 4 h.

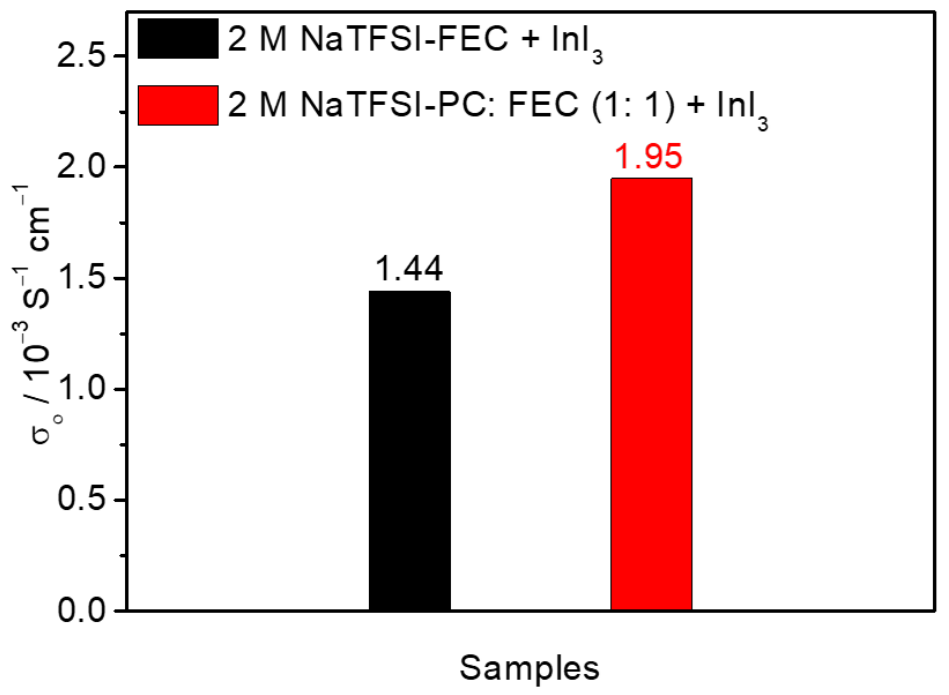

Supplementary Figure 11 The ionic conductivity values of 2 M NaTFSI in FEC with 10 mM InI_3 electrolyte and 2 M NaTFSI in PC: FEC (1: 1 by volume) with 10 mM InI_3 electrolyte at 25 ^{o}C.

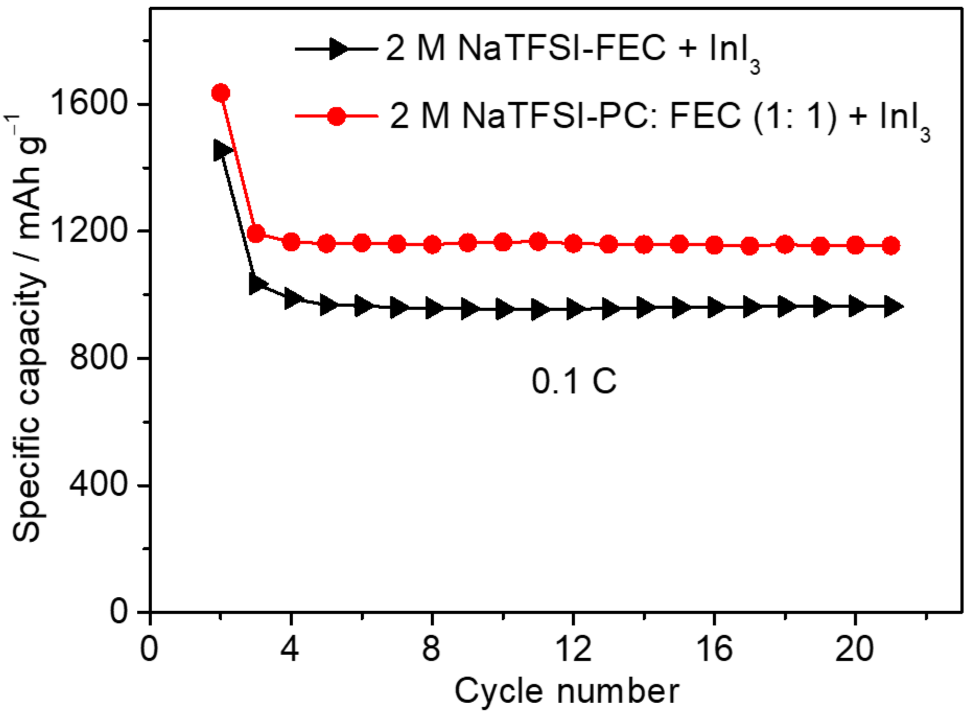

Supplementary Figure 12 Cycling performances of Na/S@MPCF cells with 2 M NaTFSI in FEC with 10 mM InI_3 electrolyte and 2 M NaTFSI in PC: FEC (1: 1 by volume) with 10 mM InI_3 electrolyte at 0.1 C.

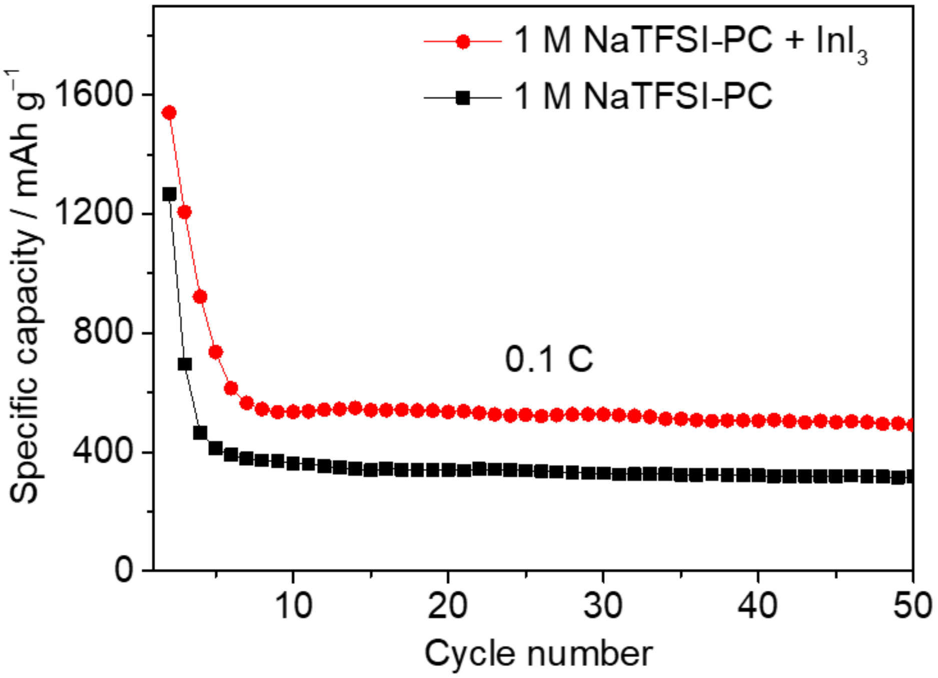

Supplementary Figure 13 Cycling performances of Na/S@MPCF cells using 1 M NaTFSI in PC with 10 mM InI_3 and 1 M NaTFSI in PC electrolytes at 0.1 C. With the addition of InI_3, the initial discharge capacity increases from 1268 mAh g^{-1} to 1541 mAh g^{-1}, and stabilizes at 481 mAh g^{-1} after 50 cycles at 0.1 C.

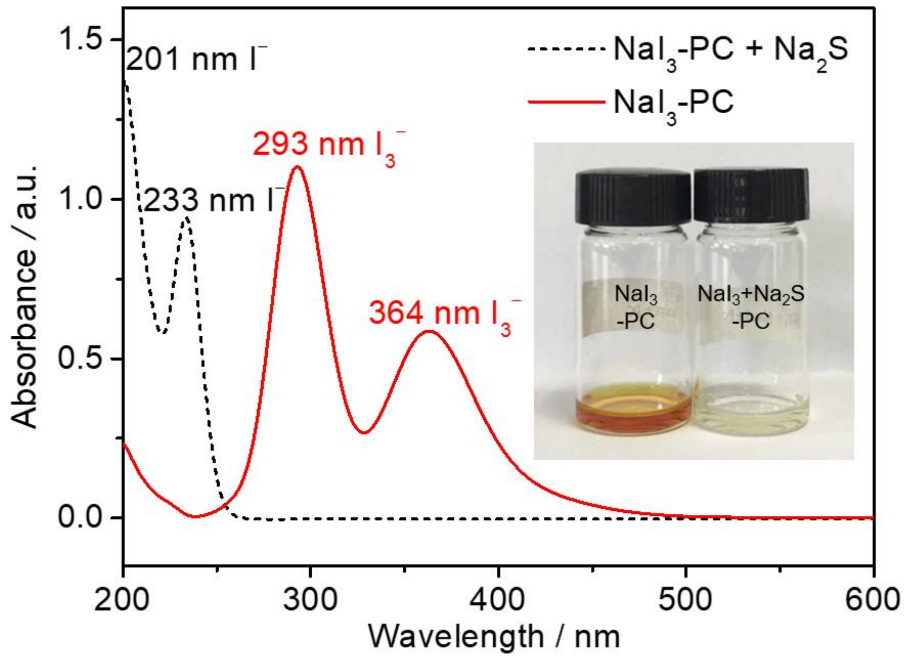

Supplementary Figure 14 UV-Vis spectra of 0.8 mg NaI_3 in 1 mL PC, and 0.8 mg NaI_3 with 0.3 mg Na_2S in 1 mL PC after aging for 0.5 h at room temperature. The corresponding optical images are shown in the inset.

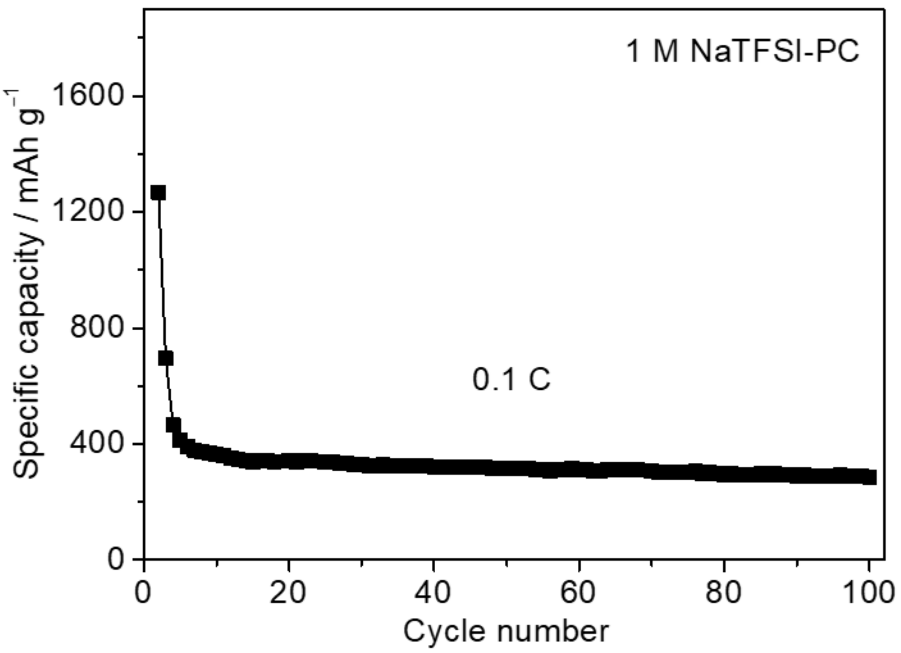

Supplementary Figure 15 Cycling performance of Na/S@MPCF cell with 1 M NaTFSI in PC electrolyte at a current density of 0.1 C.

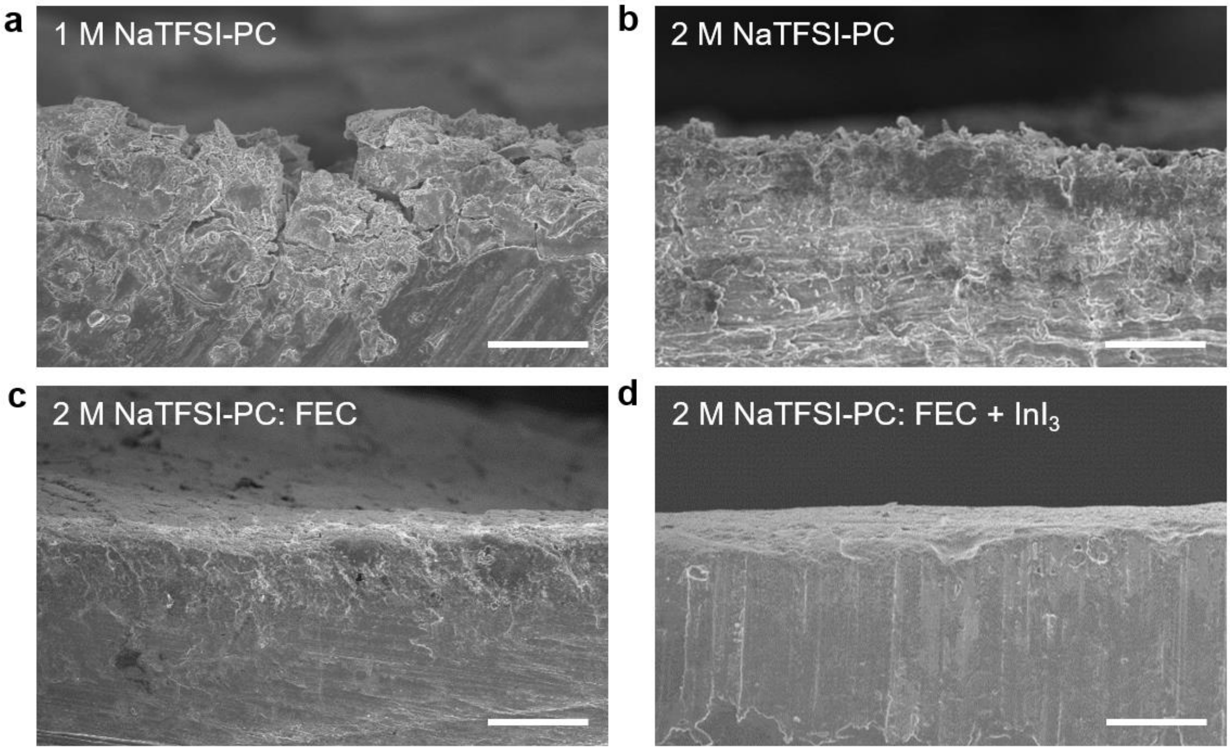

Supplementary Figure 16 Cross-sectional FE-SEM images of Na anodes obtained from Na/S@MPCF cells using different electrolytes after 50 cycles at 0.1 C, corresponding to Fig. 2e–h in the manuscript. Scale bars are 50 μm in Supplementary Fig. 16a–d.

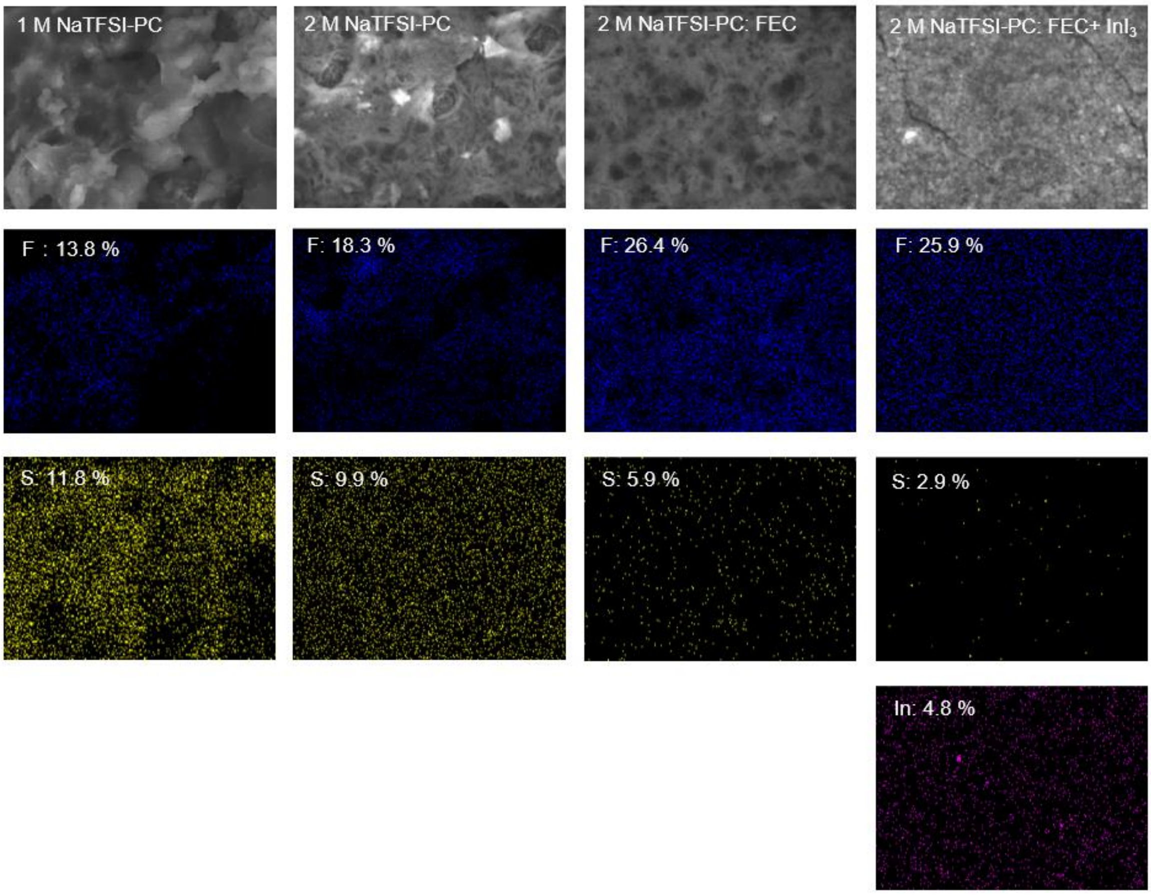

Supplementary Figure 17 EDS mappings of F, S and In elements on the Na anodes obtained from Na/S@MPCF cells using different electrolytes after 50 cycles at 0.1 C, corresponding to Fig. 2 e–h in the manuscript.

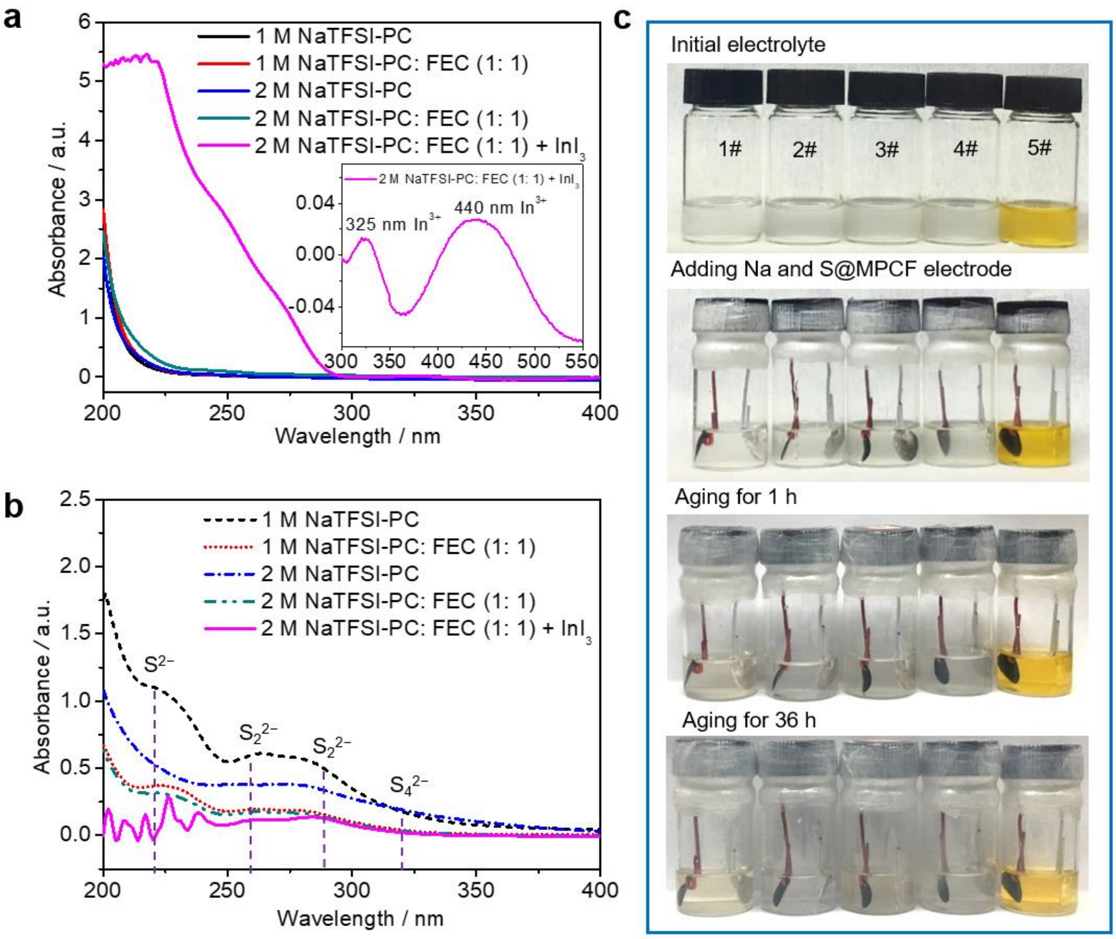

Supplementary Figure 18 The formation Na polysulfides in different electrolytes. UV-Vis spectra of **a** the five initial electrolyte samples and **b** electrolyte samples with same amount of sulfur powder and Na metal foils after aging at 60 ^{o}C for 36 h, corresponding to Fig. 3b. **c** Visual observation of Na polysulfides formation in five electrolyte samples (1#:1 M NaTFSI in PC; 2#: 1 M NaTFSI in PC: FEC (1: 1 by volume); 3#: 2 M NaTFSI in PC; 4#: 2 M NaTFSI in PC: FEC (1: 1 by volume); 5#: 2 M NaTFSI in PC: FEC (1: 1 by volume) with 10 mM InI_3) along with aging time at 60 ^{o}C. The S@MPCF electrodes with sulfur loading of ~1.2 mg cm^{-2} and Na metal foils were added into the electrolytes to simulate the self-discharge processes.

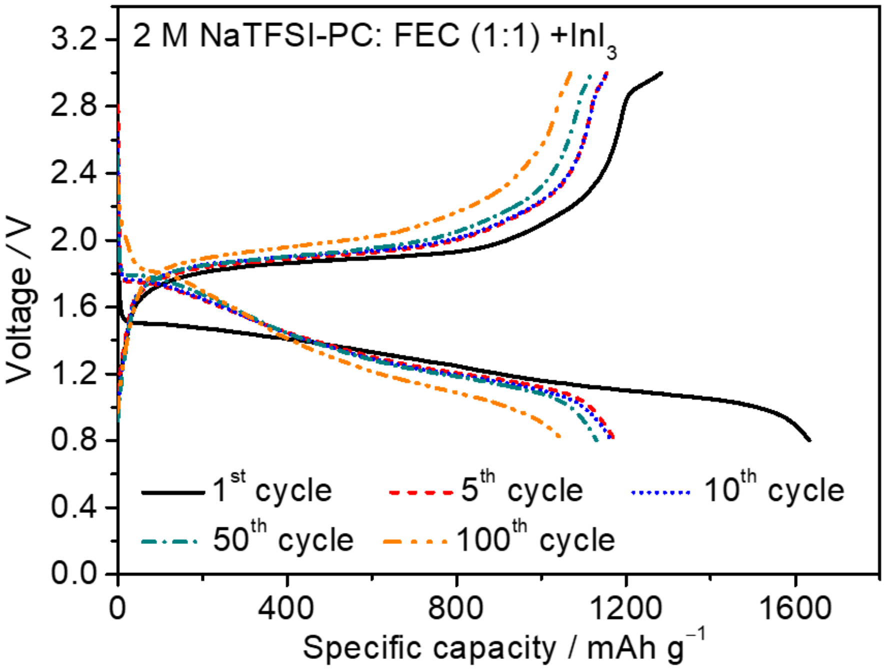

Supplementary Figure 19 The 1st, 5th, 10th, 50th and 100th charge/discharge profiles of Na/2 M NaTFSI in PC: FEC (1: 1 by volume) with 10 mM InI_3/S@MPCF cell at 0.1 C.

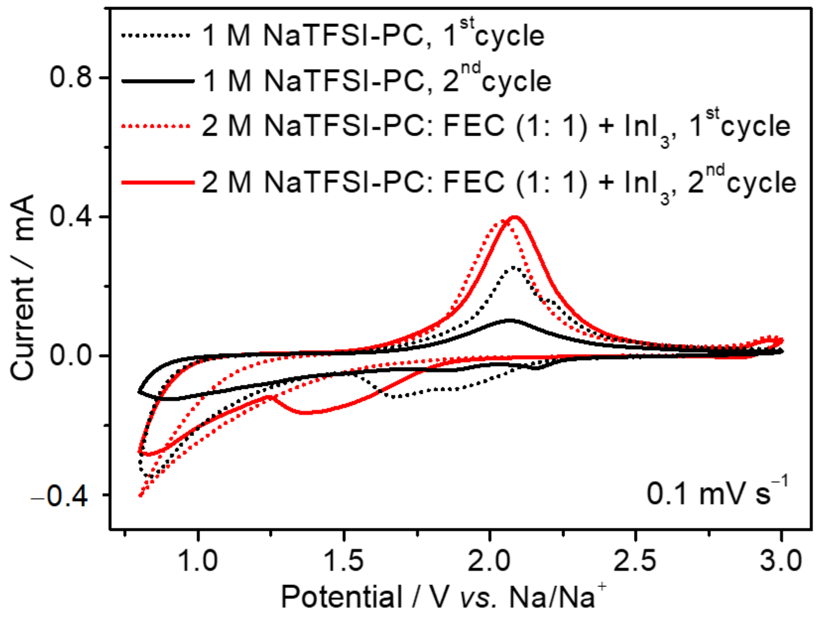

Supplementary Figure 20 CV curves of Na/S@MPCF cells using1 M NaTFSI in PC and 2 M NaTFSI in PC: FEC (1: 1 by volume) with 10 mM InI_3 electrolytes at 0.1 mV s^{-1}.

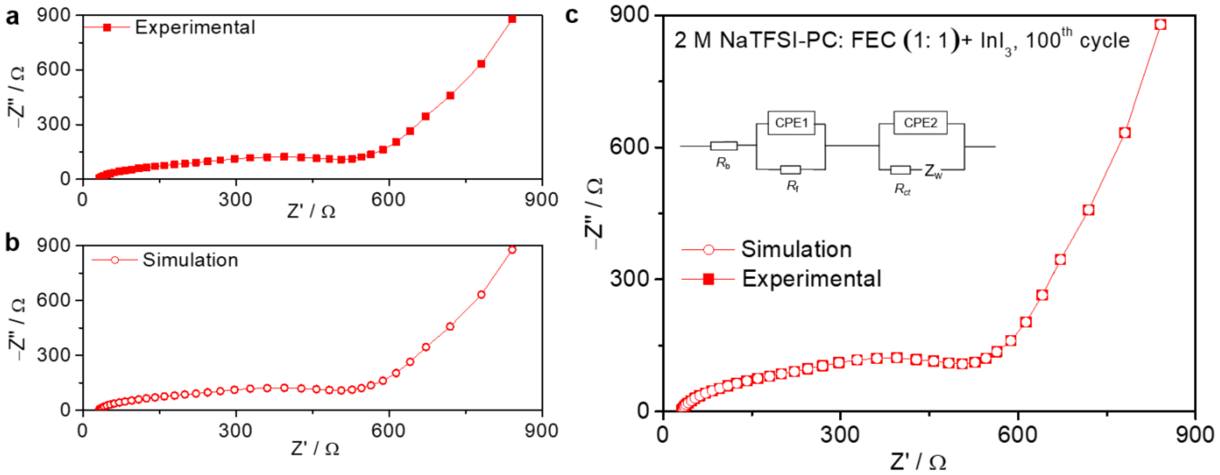

Supplementary Figure 21 Typical **a** experimental, **b** simulation EIS curves using an equivalent circuit[2] and **c** the simulating result of the Na/2 M NaTFSI in PC: FEC (1: 1 by volume) with 10 mM InI_3/S@MPCF cell.

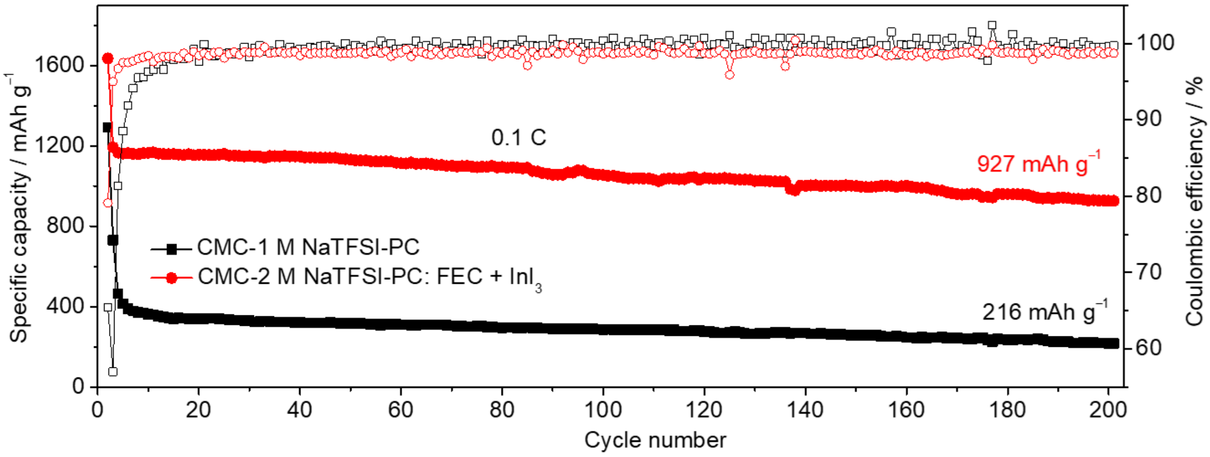

Supplementary Figure 22 Cycling performances of Na/S@MPCF cells using 1 M NaTFSI in PC and 2 M NaTFSI in PC: FEC (1: 1 by volume) with 10 mM InI_3 electrolytes at 0.1 C.

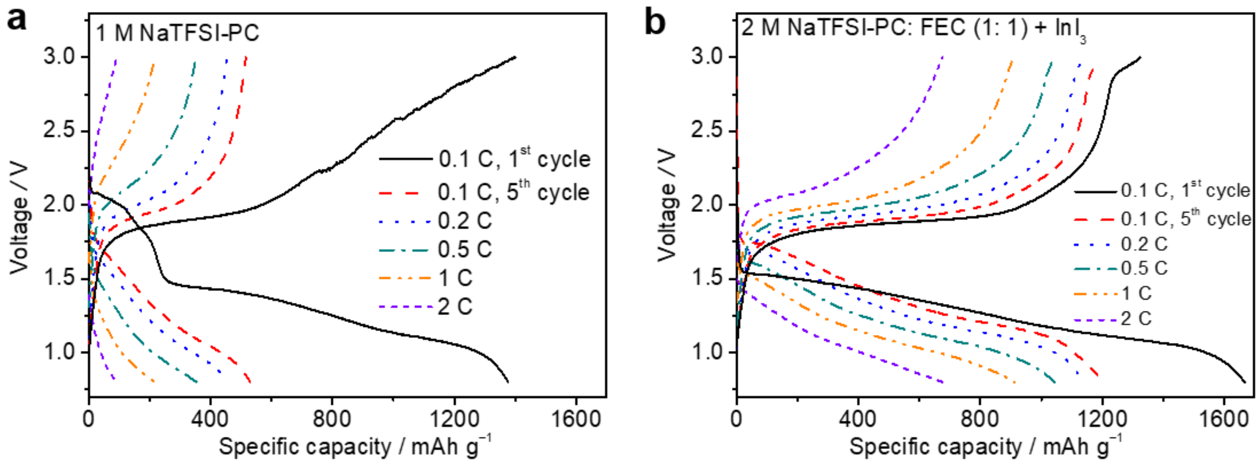

Supplementary Figure 23 The charge/discharge profiles of Na/S@MPCF batteries using electrolytes **a** 1 M NaTFSI in PC and **b** 2 M NaTFSI in PC: FEC (1: 1 by volume) with 10 mM InI_3 electrolytes at different current densities.

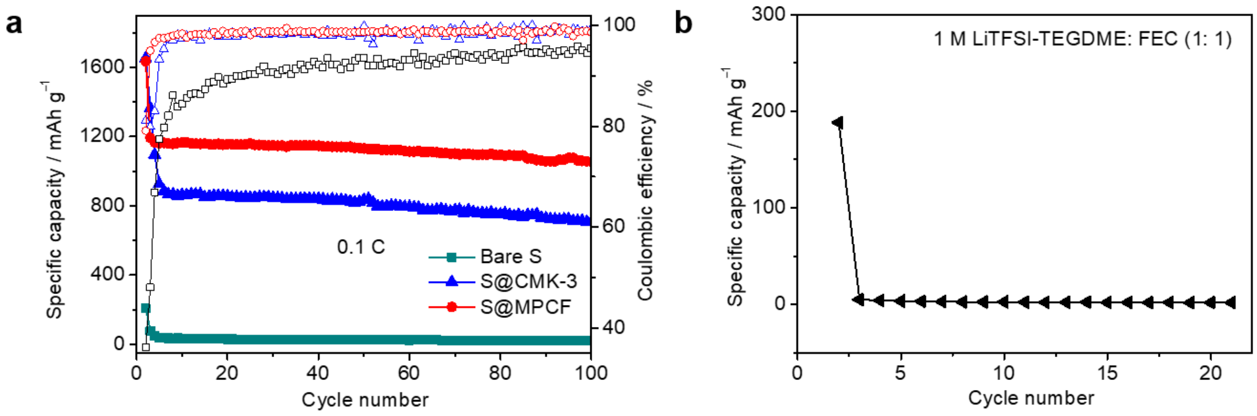

Supplementary Figure 24 The widen application of the FEC-based electrolytes. a Cycling performances of bare sulfur cathode, S@CMK3 cathode and S@MPCF in Na-S batteries using 2 M NaTFSI in PC: FEC (1: 1 by volume) with 10 mM InI_3 electrolyte at 0.1 C; **b** Cycling performances of Li/S@MPCF cell with 1 M LiTFSI in TEGDME: FEC (1: 1 by volume) electrolyte at 0.1 C.

Supplementary Tables

Supplementary Table1 A comparation of previously reported room-temperature Na-S batteries using different S@ porous carbon electrodes.

**Carbon matrix/Average pore size /Sulfur content**	**Cathode Composition**	**Electrolyte**	**Initial Capacity**	**Cycling Performances**	**References**
Carbon nanotube (CNT)@ microporous carbon (MPC)/~0.5 nm/40 wt%	S/(CNT@MPC): super-P: PVDF=8: 1: 1	1 M $NaClO_4$ -PC: EC (v: v=1: 1)	1148 mAh g^{-1} at 0.1 C	600 mAh g^{-1} at 1 C after 200 cycles	[3]
Microporous carbon (MC)/<0.7 nm/80 wt%	MC/S composite: super-P: PVDF=7: 2: 1	1 M $NaClO_4$-EC: DEC (v: v=1: 1)	~1600 mAh g^{-1} at 0.1 C	392 mAh g^{-1} at 1 C after 200 cycles	[4]
Nanoporous nitrogen doped carbonized ZIF-8 (cZIF-8)/1 nm/50 wt%	cZIF-8/S: Super-P: PVDF=7: 2: 1	1 M $NaClO_4$-TEGDME	873 mAh g^{-1} at 0.2 C	500 mAh g^{-1} at 0.2 C after 250 cycles	[5]
Ordered microporous carbon sphere/0.5 nm/35 wt%	S@C: carbon black: CMCNa =8: 1: 1	1 M $NaPF_6$-TEGDME + 0.25 M $NaNO_3$	~440 mAh g^{-1} at 1C	300 mAh g^{-1} at 1 C after 1500 cycles	[6]
Nitrogen, sulfur-doped hierarchical porous carbon (N, SHPC)/4.2 nm/66.6 wt%	N, SHPC/S: carbon black: (SBR: CMCNa) =7: 2: 1	1 M $NaClO_4$-EC: PC (v: v=1: 1)	455 mAh g^{-1} at ~0.07 C	378 mAh g^{-1} at ~0.15 C after 350 cycles	[7]
Interconnected mesoporous carbon hollow nanospheres (iMCHS)/3.6~3.8 nm/46 wt%	S@iMCHS: carbon black: CMCNa=7: 1: 2	1.0 M $NaClO_4$ -PC: EC (v: v=1: 1) + 5 wt % FEC	1215 mAh g^{-1} at ~0.06 C	292 mAh g^{-1} at ~0.06 C after 200 cycles	[8]
**Multiporous carbon fibers/2.6 nm/60 wt%**	**S@MPCF: super-P: CMCNa=8: 1: 1**	**1 M NaTFSI-PC**	**1292 mAh g^{-1} at 0.1 C**	**286 mAh g^{-1} at 0.1 C after 100 cycles**	**This work**

Supplementary Table2 The ionic conductivity values for electrolyte samples at 25 °C and the VTF fitting parameters in Fig. 2a.

Electrolyte	σ_o at 25 °C (S^{-1} cm^{-1})	σ_o (S^{-1} cm^{-1} $K^{-1/2}$)	E_a (eV)	T_o (K)
1 M NaTFSI-PC	4.76×10^{-3}	7.15	5.93×10^{-2}	144.40
1 M NaTFSI-PC: FEC (1: 1)	3.81×10^{-3}	6.40	5.56×10^{-2}	157.37
2 M NaTFSI-PC: FEC (1: 1)	1.98×10^{-3}	6.27	5.35×10^{-2}	179.24
2 M NaTFSI-PC: FEC (1: 1) + InI_3	1.95×10^{-3}	6.27	5.35×10^{-2}	179.32

Supplementary Table3 EIS simulation results from Fig. 4e.

Electrolyte type	1 M NaTFSI-PC			2 M NaTFSI-PC: FEC (1: 1) + InI_3		
Cycle number	R_b (Ω)	R_f (Ω)	R_{ct} (Ω)	R_b (Ω)	R_f (Ω)	R_{ct} (Ω)
After 3 cycles	5.82	156.2	372.3	6.48	58.9	251.4
After 100 cycles	37.11	789.2	856.2	30.94	75.1	547.5

Supplementary Notes

Supplementary Note 1. Characterization of MPCF and S@MPCF.

As seen from the SEM (Supplementary Fig. 2a) and TEM (Supplementary Fig. 2b) images of MPCFs, there are abundant pores homogeneously distributing in the fibers with a diameter of 200~300 nm. Massive mesoporous (with a pore size of about 5~10 nm) originated from the ferric acetylacetonate (FeAcAc) template appears in the MPCFs; meanwhile many micropores originated from the alkali activation process homogeneously distribute in the outer layer of MPCFs. After infusing sulfur into the MPCF, the obtained S@MPCF maintains an unbroken fiber structure (Supplementary Fig. 2c). The sulfur was uniformly distributed in the carbon matrix rich in oxygen atoms on its surface according to the elemental mapping (Supplementary Fig. 2d), and a sulfur/carbon ratio of ~1.2 is observed (Supplementary Fig. 2d, inset). The abundant multiporous pores in the MPCFs give rise to a large Brunauer-Emmett-Teller surface area of 2475 m^2 g^{-1} calculated from the N_2 adsorption-desorption isotherm (Supplementary Fig. 2e), meanwhile the pore volume of MPCF is as high as 1.6 cm^3 g^{-1} and the mean pore size is around 2.6 nm (Supplementary Fig. 2e, inset). Considering the density in molten sulfur is ~1.82 g cm^{-3} and the density of sulfur powder is 1.96~2.07 g cm^{-3} [9], the theoretical sulfur loading in MPCF is calculated to be 74.4~76.8 wt%. The sulfur loading of S@MPCF is ~61.09 wt%, verified by the TGA curve in Supplementary Fig. 2f. In this work, the temperature for the sulfur-impregnation process was firstly set as 155 ^{o}C, and then further increased to 300 °C with the purpose of infiltrating the sulfur on the surface of MPCF into the pores[10]. The cycling performance of the Na/S@MPCF cell using S@MPCF obtained by further heating the sulfur/MPCF mixture at 300 ^{o}C (labeled as “S@MPCF-300 ^{o}C”) is shown in Supplementary Fig. 2g. Compared with the S@MPCF heated at 155 ^{o}C (labeled as “S@MPCF-155 ^{o}C”), the S@MPCF-300 ^{o}C cathode shows a slightly higher initial Coulombic efficiency (79.1 % *vs.* 63.5 %) and reversible capacity after 20 cycles (1155.1 mA h g^{-1} *vs.* 1064 mA h g^{-1}), demonstrates an improvement on the utilization of sulfur.

A comparation of previously reported room-temperature Na-S batteries using different S@ porous carbon electrodes are listed in Supplementary Table 1. It is seen that the electrochemical performances of Na-S batteries are greatly affected by the pore size of the carbon host. The

electrochemical performance of the S@MPCF (with an average pore size of 2.6 nm) electrode in this work is poorer than those applying S@microporous carbon (with an average pore size of 0.5~1 nm) electrodes, but at the same level as other S@mesoporous carbon electrodes. Furthermore, it is noticed that the cycling capacities of Na-S cells using S@porous carbon electrodes and unmodified electrolytes are generally less than 600 mAh g^{-1}. Only by optimizing the electrolyte (this work) can the cycling capacity of Na-S cells be increased to >900 mAh g^{-1}.

Supplementary Note 2. The effect of different binders on the electrochemical performances.
As seen in Supplementary Fig. 3a, compared with poly(vinylidene fluoride) (PVDF), CMCNa binder significantly improves cycling performance of the S@MPCF cathode (927 mAh g^{-1} *vs.* 560 mAh g^{-1} after 200 cycles at 0.1 C). The Na-S cell with CMCNa binder delivers a specific capacity of 1170 mAh g^{-1}, 1107 mAh g^{-1}, 984 mAh g^{-1}, 867 mAh g^{-1} and 699 mAh g^{-1} at 0.1, 0.2, 0.5, 1 and 2 C, respectively, and recovers to 1140 mAh g^{-1} when the current density is switched back down to 0.1 C. This is much higher than cell using PVDF binder (Supplementary Fig. 3b). Furthermore, the potential gap between the 5th charge and discharge curves of Na-S cell with CMCNa binder is obviously smaller than that with PVDF binder, indicating a decrease in polarization (Supplementary Fig. 3c).

Such good electrochemical performance of the cell using CMCNa binder can be attributed to the formation of strong covalent S-O bond between CMCNa and sulfur. As seen from the FTIR spectra of CMCNa, the CMCNa/S composite (obtained by grinding the mixture for 20 minutes), MPCF and S@MPCF transmission bands at about ~720 and ~650 cm^{-1} assigned to S-O vibration appear in both the spectra of CMCNa/S and S@MPCF (Supplementary Fig. 3d)[11]. This demonstrates a formation of surface bonds between sulfur and CMCNa binder or the -OH group (~3400 cm^{-1}) on the surface of the MPCF matrix during the preparation process of electrode. XPS measurements (Supplementary Fig. 3e) were performed to further verify the FTIR result. These show peaks at about 530.5 eV in O1s spectra and at around 166 eV in S 2p spectra of CMCNa/S and S@MPCF, which are attributed to S-O bond[12,13] that can greatly reduce the dissolution of Na polysulfides and therefore enhance the performance of Na-S batteries[14].

Supplementary Note 3. Electrochemical characterization of Na-S and Li-S batteries in

TEGDME-based and PC-based electrolytes.

As shown in Supplementary Fig. 4a, Li/S@MPCF cells deliver a discharge capacity of 521 mAh g^{-1} after 20 cycles in 1 M LiTFSI-TEGDME electrolyte at 0.1 C, whereas the cells hardly charge and discharge in 1 M LiTFSI-PC electrolyte (only 1 mAh g^{-1} after 20 cycles at 0.1 C). Compared with above Li-S battery system, Na/S@MPCF cell suffers from a serious capacity fading in 1 M NaTFSI-TEGDME electrolyte (638 mAh g^{-1} and 145 mAh g^{-1} in the 1st and 20th cycle at 0.1 C, respectively). On the contrary, Na/S@MPCF cells maintained a much higher discharge capacity of 349 mAh g^{-1} in 1M NaTFSI-PC electrolyte after 20 cycles at 0.1 C (Supplementary Fig. 4b and Supplementary Fig. 5).

Such distinct performance variation can be explained as follows: (1) The stability of solvents towards Na anodes. It is seen from the galvanostatic cycling measurement in Supplementary Fig. 4c that the Li/Li symmetric cell using 1 M LiTFSI-PC electrolyte displays a smaller overpotential compared with that using 1 M LiTFSI-TEGDME electrolyte during 100 h cycles, which is mainly caused by a thicker solid electrolyte interface (SEI) and less stable electrolyte/electrode interface (caused by nonuniform Li deposition and dendrite growth[15]) in TEGDME-based electrolytes than those in PC-based electrolytes[16]. Furthermore, in the Na/Na symmetric cells, a high voltage fluctuation associated with short-circuits caused by dendrite growth (after about 30 h) is observed when using 1 M NaTFSI-TEGDME electrolyte (Supplementary Fig. 4d). This may be attributed to the more serious interfacial side reactions between Na metal and TEGDME due to the stronger metallicity of Na than Li. The Na/Na cell using 1 M NaTFSI-PC electrolyte, however, keeps a stable voltage hysteresis at a current density of 0.1 mA cm^{-2} for 100 h cycles, indicating a relatively uniform Na growth with stable a Na/PC-based electrolyte interface. Such relatively

good compatibility between PC-based electrolyte and Na is quite important to the long-term cycling of Na-S batteries. (2) The solubility of Na polysulfides in solvents. Supplementary Fig. 4e displays the photographs of Na polysulfides solutions, which were prepared by adding same amounts of Na_2S and sulfur (1: 7 by molar ratio) into both TEGDME and PC solvents. After aging for 10 h, the color of the PC solution shows as light yellow meanwhile that with TEGDME turns brown, validating that the PC solvent has a much lower solubility for high-order Na_2S_x (x = 5~8, which have dark color[17]) than TEGDME.

Supplementary Note 4. The reactions between polysulfide intermediates and carbonate solvents.

It is well known that in Li-S batteries, the nucleophilic sulfide anions actively react with carbonate solvents via nucleophilic addition or substitution reaction, which results in a rapid capacity fading[18]. As shown in Supplementary Fig. 6a, same amounts of sulfur (10 mg) together with Li or Na foils were added into different electrolytes to observe the formation of polysulfides. After aging for 48 h, the TEGDME-based electrolyte immersing with Li foil obviously turned to dark brown color due to the formation of Li polysulfides; meanwhile the PC-based electrolyte immersing with Li foil almost did not change its color, which indicates that Li polysulfides cannot be massively formed in carbonate-based electrolytes due to side reactions. This can be further verified by the images of separators (Supplementary Fig. 6b**)** together with the Raman spectra of sulfur electrodes (Supplementary Fig. 6c) obtained from discharged Li-S cells, and also the UV-Vis spectra results (Supplementary Fig. 6d). In sharp contrast, it is seen from Supplementary Fig. 6a and Supplementary Fig. 6b that dark-colored Na polysulfides can be generated in PC-based electrolyte, meanwhile the Raman spectrum of discharged sulfur electrode

from Na/1 M NaTFSI in PC/bare S cell shows clear peaks of Na polysulfids and Na_2S (Supplementary Fig. 6c)[19]. This may be due to the fact that the larger ionic radius of Na^+ than Li^+ leads to less dissociation in polar solvents, which results in a lower reactivity of Na^+-polysulfide$^-$ ion pairs than that of Li^+-polysulfide$^-$ ion pairs[20,21]. Hence, the side reactions between Na polysulfides and carbonate solvents are much less severe than those between Li polysulfides and carbonate solvents.

As shown in Supplementary Fig. 6e, bare sulfur electrodes without surface coating do not exhibit any noticeable capacity in Li-S or Na-S batteries with PC-based electrolyte during cycling. When composited with porous carbon, S@MPCF electrodes can maintain a relatively stable capacity (349 mAh g^{-1} after 20 cycles at 0.1 C) in Na-S cells with PC-based electrolyte, but in Li-S cells it was observed to still deliver only 1 mAh g^{-1} capacity after 20 cycles at 0.1 C with PC-based electrolyte. This phenomenon can be explained as follows. As shown in the CV curves in Supplementary Fig. 6h, a strong peak related to the formation of Na polysulfides appears in the cathodic scan of Na/1 M NaTFSI in PC/bare S cell, corresponding a large initial discharge capacity of 828 mAh g^{-1} (Supplementary Fig. 6g). These are obviously different from the Li/1 M LiTFSI in PC/base S cells with a quite weak reduction peak (Supplementary Fig. 6h) and a very limited capacity (169 mAh g^{-1}, Supplementary Fig. 6f). This verifies that the side reactions between Na polysulfides and carbonate solvents is negligible as mentioned before. However, for the Na/1 M NaTFSI in PC/S cell, no oxidation peak is observed in the subsequent anodic scan (Supplementary Fig. 6h), meanwhile the discharge capacity is irreversible in the following charging process (Supplementary Fig. 6e). This is probably because the volume change (~260 %) during the cathodic reaction from sulfur to Na_2S is much higher that the volume change from

sulfur to Li_2S (~80 %)[6]. As seen from Supplementary Fig. 6k, after the initial discharging process, cracks and holes caused by the such huge volume expansion appear on the surface of the bare sulfur electrode, which lead to an irreversible loss of active material and a rapid capacity fading in the following charging. Therefore, it is necessary to immobilize sulfur inside of carbon matrix to relieve the stress caused by the volume change, which gives rise to the preferable cycling performance for the Na/PC-based electrolyte/S@MPCF cells.

Supplementary Note 5. The formation of Na polysulfide in solvents with different FEC proportions.

Supplementary Fig. 10a displays the color changes for various volume proportions of FEC in PC solvent (0 % (pure PC solvent), 5 %, 10 %, 50 % and 100 % (pure FEC solvent)) with the addition of Na_2S and sulfur in a molar ratio of 1: 7. After aging at 60 ^oC for 4 h, pure PC solvent became dark-brown due to the formation of soluble polysulfides. However, the color of solvent remains light yellow or transparent with increasing FEC proportion, clearly indicating the insoluble behavior of Na polysulfides in FEC solvent.

For further clarification, UV-Visible spectrophotometry was applied to the five mixture solutions. As shown in Supplementary Fig. 10c, various Na polysulfides (S_6^{2-} at 480 nm, S_4^{2-} at 410 and 320 nm, S^{2-} and S_2^{2-} at 220~260 nm[1,22,23]) present in the UV-Vis spectrum of pure PC/Na_2S_8 mixture after aging at 60 ^oC for 5 h compared with the baseline of initial solvents in Supplementary Fig. 10b. However, the corresponding peak intensities of various Na polysulfides gradually decrease with the increase in FEC proportion, which is well consistent with the color change in Supplementary Fig. 10a and demonstrates the low solubility of polysulfides in FEC.

Supplementary Note 6. Verification of the reaction between I_3^- and Na_2S.

The UV-Vis spectra in Supplementary Fig. 14 give clear evidences for the transformation from I_3^- (corresponding bands at 293 nm and 364 nm[24]) to I^- (corresponding bands at 201 nm and 233 nm[24]) after the addition of Na_2S. The color changes shown in inset also confirm the disappearance of dark-brown I_3^- and Na_2S powder. This phenomenon verifies the reaction

between I_3^- and Na_2S shown in equation (4).

Supplementary Note 7. The formation Na polysulfides in different electrolytes.

Supplementary Fig. 18a exhibits the UV-Vis spectra of the five initial electrolyte samples, which are employed as baselines for the corresponding samples with sulfur and Na metal foils shown in Supplementary Fig. 18b. The initial 2 M NaTFSI in PC: FEC (1: 1 by volume) with 10 mM InI_3 electrolyte presents a low transparency due to the yellow In^{3+} ion (corresponding bands at 325 nm and 440 nm[25,26], inset of Supplementary Fig. 18a) in it. After adding sulfur powder and Na metal foil into the solvents, various Na polysulfides appear with increasing aging time. The intensity of bands related to Na polysulfides in Supplementary Fig. 18b is well coincident with the tendencies of color variation in Fig. 3b (1 M NaTFSI in PC > 2 M NaTFSI in PC > 1 M NaTFSI in PC: FEC (1: 1 by volume) > 2 M NaTFSI in PC: FEC (1: 1 by volume) > 2 M NaTFSI in PC: FEC (1: 1 by volume) with 10 mM InI_3). We have further performed a self-discharge experiment using S@MPCF electrodes instead of sulfur powder. The phenomenon in Supplementary Fig. 18c is basically in consistent with the previous self-discharge experiment using sulfur powder (Fig. 3b). However, the changes in color are less obvious due to the confinement of sulfur in the MPCF porous carbon.

Supplementary Note 8. Description on the EIS simulation.

As can be seen from Supplementary Fig. 21, the experimental and simulated spectra are quite well matched. According to the equivalent circuit, the intersection of the diagram with the real axis refers to a bulk resistance (R_b), reflecting the resistance of electrodes and electrolyte/separator. The depressed semicircle at high frequency can be attributed to the interfacial resistance (R_f) and *CPE1*, while the depressed semicircle at medium frequency can be ascribed to the charge transfer resistance (R_{ct}) and *CPE2*. Instead of the capacitance of the passivation layer (C_f) and double-layer capacitance (C_{dl}), *CPE1* and *CPE2* are the constant phase elements used to take the roughness of the particle surface into account. The line at low frequency is equivalent to the Warburg impedance (Z_w), which is related to the sodium ion diffusion within the particles[2].

Supplementary Note 9. The widen application of the FEC-based electrolytes.

It is interesting that a high proportion of FEC in electrolyte greatly enhances the electrochemical performance of Na-S cells, but significantly deteriorates the electrochemical performance of Li-S cells (Supplementary Fig. 24b), mainly due to the side reaction between polysulfide intermediates and carbonate solvent as mentioned before.

Supplementary Methods

Preparation and characterization of multiporous carbon fibers (MPCFs). Carbonized polyacrylonitrile (PAN) based nanofibers were activated by potassium hydroxide (KOH) to obtain the MPCFs. As shown in Supplementary Fig. 1, the electrospinning solution was prepared by dissolving 0.6 g PAN (Mw= 150000, Sigma Aldrich), 0.3 g polymethyl methacrylate (PMMA, Mw= 350000, Sigma Aldrich) and 0.6 g ferric acetylacetonate (FeAcAc, Aladdin) in 12 mL N, N-dimethylformamide (DMF, Macklin) with stirring at 70 ^{o}C for 5 h. As a typical procedure for electrospinning, the voltage and the feeding rate were fixed at 20 kV and 1.5 mL h^{-1}, respectively. The needle-to-collector distance was fixed at 18 cm. Subsequently, the sample was pre-oxidized at 220 ^{o}C for 2 h in air with a heating rate of 10 ^{o}C min^{-1}, and then further carbonized at 700 ^{o}C for 1 h in argon (Ar) with a heating rate of 5 ^{o}C min^{-1} to obtain carbonized nanofibers (CFs). Then the CFs were washed with 3 M hydrochloric acid (HCl, Sinopharm) solution at 60 ^{o}C for 5 h to remove the Fe_3O_4 inside. The activation process of CFs were carried out with a dry mixture of KOH (Aladdin) and CFs (mass ratio =3: 1) at 700 ^{o}C for 1 h under flowing Ar. After that, the resultant mixture was washed with 3 M HCl solution followed by deionized water at room temperature. The powders were dried at 60 ^{o}C under vacuum for 12 h to obtain the final MPCFs.

Field emission scanning electron microscope (FE-SEM, HITACHS4800) and high-resolution transmission electron microscopy (HR-TEM, FEI TECNAIG2 F30) were carried out to investigate the morphology of materials. The specific surface areas were measured via the Brunauer-Emmett-Teller (BET) method at 77 K with N_2 as analysis gas. The pore size distribution of MPCFs was calculated from the adsorption branch of the isotherms based on the Barrett-Joyner-Halenda (BJH) model.

Construction and characterization of S@CMK3 and bare sulfur electrodes. S@CMK-3 was obtained via CMK-3 mesoporous carbon (XF Nano, Nanjing) and nano sulfur powder at a weight ratio of 4: 6 in the same way. The thermogravimetric analyses (TGA) of S@MPCF and S@CMK-3 composites were performed using the TGA Q5000 IR thermogravimetric analyzer under a pure N_2 flow at a rate of 10 ^{o}C min^{-1}.

The S@CMK-3 electrodes were prepared by following the same procedure, meanwhile bare sulfur electrodes were composed of 48 wt% sulfur, 42 wt% Super-P and 10 wt% CMCNa. Poly(vinylidene fluoride) (PVDF) and N-methyl-2-pyrrolidone (NMP) were used as the alternative binder and dispersant for comparison, respectively. In S@MPCF cathodes, the mass loading of sulfur was set at about 0.40, 1.40, 1.77, 4.83 and 5.24 mg cm^{-2} respectively for the tests. Fourier transform infrared spectroscopy (FTIR, Thermo Scientific Nicolet iS 50) and X-ray photoelectron spectroscopy (XPS, Physical Electronics PHI5802) were used to analyze the electrodes. In the XPS test, the C 1s region was used as references and set at 284.8 eV.

Assembly and characterization of Li-S batteries. CR2032 coin cells were assembled in an Ar-filled glove box using Celgard 2500 film as the separator for Li-S batteries. The sulfur/electrolyte ratio in each cell was uniformly set at ~50 g L^{-1}. The assembled Li-S cells were cycled between 1.7 and 2.8 V at various charge/discharge rates (1 C = 1675 mA g_S^{-1}) on a Land 2001 A battery testing system at 25 ^{o}C.

Supplementary References

1 Manan, N. S. *et al.* Electrochemistry of sulfur and polysulfides in ionic liquids. *J. Phys. Chem. B* **115**, 13873−13879 (2011).

2 Liu, M. *et al.* Novel gel polymer electrolyte for high-performance lithium-sulfur batteries. *Nano Energy* **22**, 278−289 (2016).

3 Xin, S. *et al.* A high-energy room-temperature sodium-sulfur battery. *Adv. Mater.* **26**, 1261−1265 (2014).

4 Hu, L. *et al.* Ultramicroporous carbon through an activation-free approach for Li-S and Na-S batteries in carbonate-based electrolyte. *ACS Appl. Mater. Interf.* **9**, 13813−13818 (2017).

5 Chen, Y.-M. *et al.* A nitrogen doped carbonized metal-organic framework for high stability room temperature sodium-sulfur batteries. *J. Mater. Chem. A* **4**, 12471−12478 (2016).

6 Carter, R. *et al.* A sugar-derived room-temperature sodium sulfur battery with long term cycling stability. *Nano Lett.* **17**, 1863−1869 (2017).

7 Qiang, Z. *et al.* Ultra-long cycle life, low-cost room temperature sodium-sulfur batteries enabled by highly doped (N,S) nanoporous carbons. *Nano Energy* **32**, 59−66 (2017).

8 Wang, Y. X. *et al.* Achieving high-performance room-temperature sodium-sulfur batteries with S@interconnected mesoporous carbon hollow nanospheres. *J. Am. Chem. Soc.***138**, 16576−16579 (2016).

9 Ji, X. *et al.* A highly ordered nanostructured carbon-sulphur cathode for lithium-sulphur batteries. *Nat. Mater.* **8**, 500−506 (2009).

10 Guo, J. *et al.* Sulfur-impregnated disordered carbon nanotubes cathode for lithium-sulfur batteries. *Nano Lett.* **11**, 4288−4294 (2011).

11 Ulic, S. E. *et al.* Trifluoroacetylsulfenyl trifluoroacetate, $CF_3C(O)$-S-O-$C(O)CF_3$, a novel compound with a symmetrically substituted S-O bond: synthesis, spectroscopic characterization, and quantum chemical calculations. *Inorg. Chem.* **41**, 5699−5705 (2002).

12 Urban, N. R. *et al*. Addition of sulfur to organic matter during early diagenesis of lake sediments. *Geochim. Cosmochim. Acta* **63**, 837−853 (1999).

13 Li, R. *et al.* One-step synthesis of 3D sulfur/nitrogen dual-doped graphene supported nano silicon as anode for Li-ion batteries. *Appl. Surf. Sci.* **433**, 367−373 (2018).

14 Fan, L. *et al*. Covalent sulfur for advanced room temperature sodium-sulfur batteries. *Nano Energy* **28**, 304−310 (2016).

15 Zhou, D. *et al.* SiO_2 hollow nanosphere-based composite solid electrolyte for lithium metal batteries to suppress lithium dendrite growth and enhance cycle life. *Adv. Energ. Mater.* **6**, 1502214 (2016).

16 Song, J. H. *et al.* Effect of fluoroethylene carbonate on electrochemical performances of lithium electrodes and lithium-sulfur batteries. *J. Electrochem. Soc.* **160**, A873−A881 (2013).

17 Kim, I. *et al.* Sodium polysulfides during charge/discharge of the room-temperature Na/S battery using TEGDME electrolyte. *J. Electrochem. Soc.* **163**, A611−A616 (2016).

18 Gao, J. *et al*. Effects of liquid electrolytes on the charge-discharge performance of rechargeable lithium/sulfur batteries: electrochemical and in-Situ X-ray absorption

spectroscopic studies. *J. Phys. Chem. C* **115**, 25132−25137 (2011).
19 Yeon, J. T. *et al.* Raman spectroscopic and X-ray diffraction studies of sulfur composite electrodes during discharge and charge. *J. Electrochem. Soc.* **159**, A1308−A1314 (2012).
20 Bhattacharyya D. N. *et al*. Reactivities and conductivities of ions and ion pairs in polymerization processes. *J. Phys. Chem.* **9**, 612−623 (1965).
21 Bhattacharyya, D. N. *et al*. Studies of ions and ion pairs in tetrahydrofuran solution. alkali metal salts of tetraphenylboride. *J. Phys. Chem.* **69**, 608−611 (1965).
22 Liu, M. *et al.* An efficient Li_2S-based lithium-ion sulfur battery realized by a bifunctional electrolyte additive. *Nano Energy* **40**, 240−247 (2017).
23 Zou, Q. *et al*. Solvent-dictated lithium sulfur redox reactions: an operando UV-vis spectroscopic study. *J. Phys. Chem. Lett.* **7**, 1518−1525 (2016).
24 James, M. G. *et al*. Visible light generation of iodine atoms and I-I bonds: aensitized I^- oxidation and I_3^- photodissociation. *J. Am. Chem. Soc.* **131**, 16206−16214 (2009).
25 Lassauque, N. *et al*. Nickel-catalyzed asymmetric hydrovinylation using lewis acid activation. *Eur. J. Org. Chem.* **2009**, 3199−3202 (2009).
26 Kuo, T. R. *et al.* Green synthesis of InP/ZnS core/shell quantum dots for application in heavy-metal-free light-emitting diodes. *Nanoscale Res. Lett.* **12**, 537 (2017).
